# The Impact of Bariatric Surgery on Psychological Health

**DOI:** 10.1155/2013/837989

**Published:** 2013-03-28

**Authors:** Jeremy F. Kubik, Richdeep S. Gill, Michael Laffin, Shahzeer Karmali

**Affiliations:** ^1^Faculty of Medicine and Dentistry, University of Alberta, Edmonton, AB, Canada T5H 3V9; ^2^Department of Surgery, University of Alberta, Edmonton, AB, Canada T5H 3V9; ^3^Royal Alexandra Hospital, Room 405, Community Services Center 10240 Kingsway, Edmonton, AB, Canada T5H 3V9

## Abstract

Obesity is associated with a relatively high prevalence of psychopathological conditions, which may have a significant negative impact on the quality of life. Bariatric surgery is an effective intervention in the morbidly obese to achieve marked weight loss and improve physical comorbidities, yet its impact on psychological health has yet to be determined. A review of the literature identified a trend suggesting improvements in psychological health after bariatric surgery. Majority of mental health gain is likely attributed to weight loss and resultant gains in body image, self-esteem, and self-concept; however, other important factors contributing to postoperative mental health include a patient's sense of taking control of his/her life and support from health care staff. Preoperative psychological health also plays an important role. In addition, the literature suggests similar benefit in the obese pediatric population. However, not all patients report psychological benefits after bariatric surgery. Some patients continue to struggle with weight loss, maintenance and regain, and resulting body image dissatisfaction. Severe preoperative psychopathology and patient expectation that life will dramatically change after surgery can also negatively impact psychological health after surgery. The health care team must address these issues in the perioperative period to maximize mental health gains after surgery.

## 1. Introduction

Obesity, as defined by a body mass index (BMI) > 30 kg/m^2^, is a chronic disease that is increasing in prevalence in adults, adolescents, and children. It has been described by the World Health Organization as a global epidemic [1]. In addition, obesity is a significant risk factor for numerous comorbidities, including heart disease, diabetes, hypertension, dyslipidemia, stroke, atherosclerosis, and specific types of cancer [2]. It is also associated with overall increased mortality and decrease in lifespan by ten years [3]. Obese individuals have an increased risk of psychological distress, disordered eating, and impaired health-related quality of life (HRQoL). As the severity of the obesity rises, so does the severity of the medical complications and the mortality risk. This is important because extreme or morbid obesity, defined as BMI > 40 kg/m^2^, is one of the most rapidly growing subgroups of obesity.

Weight loss of 5 to 10% has been associated with significant reductions in comorbidities and mortality [4]. These numbers can be achieved through conventional lifestyle and pharmacologic interventions for the mild to moderately obese; however, such interventions are quite limited in morbid obesity. Currently, bariatric surgery has been shown in the literature to be an effective treatment for morbid obesity as part of an overall weight management strategy. While the efficacy of this treatment modality is often expressed as postoperative weight loss, an important and often overlooked outcome is an evaluation of the impact of surgery on psychological health. This is an important outcome measure, as it may contribute to the patient's overall concept of wellbeing. This review assesses the literature on the impact of bariatric surgery on psychological functioning of morbidly obese patients. 

## 2. Methods

 A systematic review of the current literature was conducted to evaluate the impact of bariatric surgery on the psychological health of obese patients. The search for relevant articles was conducted using both MEDLINE and PubMed databases with the following search terms: morbid obesity, bariatric surgery, psychology, psychological health, and mental health. Studies were not discriminated based on the type of bariatric procedure performed as all methods appear to be effective in treating morbid obesity. Relevant pediatric studies were also reviewed. A total of 27 articles were evaluated, including 6 literature reviews, 2 systematic reviews, and 19 primary studies. 

## 3. Results and Discussion

### 3.1. Psychological Health in Obese Individuals

A high prevalence of psychological comorbidities exists in obese patients, particularly mood disorders, anxiety, and low self-esteem. Extremely obese individuals are almost 5 times more likely than their average weight counterparts to have suffered from a major depressive episode in the past year [5]. The correlation between these two conditions is multifactorial. Body image dissatisfaction, commonly seen in obese patients, is heavily correlated with symptoms of depression [6], and this is particularly true in women, likely secondary to societal emphasis on the female physique. Obese individuals are also subjected to prejudice and discrimination, which is likely to cause or aggravate depression [7, 8]. Several studies have shown that these individuals have lower household incomes, struggle to find higher education, and are less likely to be married as compared to their nonobese peers of similar intellectual aptitude. Furthermore, repeated failed attempts to lose weight are common in this population and are likely to aggravate depressive illness, hopelessness, and poor self-esteem [9], perhaps contributing to further weight gain. Interestingly, 25–30% of bariatric patients report depressive symptoms at the time of surgery and up to 50% report a lifetime history of depression [5]. 

Similar to other obesity-related complications, psychological health appears to worsen with the increasing severity of obesity. Bariatric patients seeking surgery have a higher prevalence of psychological distress compared to other obese patients who do not seek surgery [10]. They are often driven to pursue surgery due to a distressing event. One study found that 38% of patients met diagnostic criteria of at least one Diagnostic and Statistical Manual of Mental Disorders- (DSM-) IV axis I disorder and 29% met criteria for one or more DSM-IV axis II disorders at the time of preoperative evaluation [11]. Another study suggests that obese individuals seeking medical treatment for obesity (pharmacotherapy or surgery) are more likely to suffer from psychological distress compared to their peers of similar BMI who seek behavioral therapy and dietary restriction [12]. Finally, the deterioration of psychological health in the obese population has also been attributed to the development of obesity-related comorbidities such as cardiovascular disease and type 2 diabetes mellitus [13–15]. 

### 3.2. Bariatric Surgery for Weight Loss

Mild-to-moderate obesity (BMI 30–40 kg/m^2^) may demonstrate some improvement in the short-to-medium term with lifestyle changes (dieting and exercise) and behavior therapy. As these treatments are typically ineffective for severe obesity, the literature suggests that bariatric surgery is the most effective treatment for weight loss and maintenance in the morbidly obese individual. In order to be eligible for surgery, the patient must have failed previous nonsurgical weight loss measures and either have a BMI > 40 or have a BMI > 35 in the presence of severe obesity-related comorbidity. The primary goal of bariatric surgery is for the patient to not only lose weight, but to also maintain the loss. A secondary goal is for the patients to change their eating behavior and engage in frequent exercise to promote a greater lifestyle change.

Bariatric procedures work by restricting the amount of oral intake and/or causing malabsorption. The primary bariatric procedures include laparoscopic adjustable gastric banding (LAGB), which is purely restrictive, whereas Roux-en-Y gastric bypass (RYGB) employs a combination of restrictive and malabsorptive approaches. Other procedures include laparoscopic sleeve gastrectomy (LSG) and biliopancreatic diversion. Bariatric procedure selection is based on a thorough evaluation of the patient's medical, psychological, and social issues.

A meta-analysis of bariatric surgery revealed that the mean percentage of excess weight loss was 47.5% for patients who underwent gastric banding and 61.6% for those who underwent gastric bypass [16]. Weight loss tends to stabilize around two years postoperatively, while small amounts of weight regain can occur in the third year [5]. More importantly, numerous studies have determined that surgical interventions lead to significant reversal of many obesity-related comorbidities, including type 2 diabetes, metabolic syndrome and adjusted long-term mortality [17–19]. Not only has bariatric surgery demonstrated significant and sustained weight loss, it is also a cost-effective intervention for severely obese patients [20]. 

### 3.3. Postoperative Psychological Health

Despite significant measurement heterogeneity evaluating the impact of weight loss surgery on psychological change, numerous studies and comprehensive reviews have reported overall postoperative improvement in depressive symptoms, self-esteem, health-related quality of life, and body image [21–24]. A prospective, well-controlled study of Swedish Obese Subjects (SOS) involving 4047 obese patients provides the best assessment of postoperative mental health change. Patients reported a significant decrease in depression and anxiety in the year after surgery compared to obese controls treated with diet and exercise counseling [25]. A systematic review of 40 studies from 1982–2002 reinforced these findings as consistent improvement of axis I psychiatric disorders of the DSM (particularly depression and anxiety) was reported postoperatively [22]. These psychological gains reflect those found in patients who have achieved weight reduction with behavioral or pharmacologic treatment. Furthermore, postoperative weight regain has been associated with increased depression [21]. Taken altogether, this suggests that psychopathology in the morbidly obese is likely attributable to their obesity as opposed to their underlying character. The magnitude of mental health gain may also be related to the amount of weight loss after surgery [25–27]. 

It is likely that postoperative improvements in psychological health can be attributed to more than just weight loss and self-concept as a result of weight loss. Mental health gains have been reported by patients who fail to lose weight and by patients within a few weeks after surgery, prior to any significant weight loss [28]. This may be attributable to patients taking an active role in changing their lives, despite still being overweight. Preoperative psychopathology also plays an important role as patients may report an inflated change in mood soon after surgery because they are relieved of the distressing event that initially prompted them to seek surgery [12].

Although overall improvements are found postoperatively, there remains a significant minority of patients who either report dissipation in mental health gains long-term after surgery or no psychological benefit at all [21, 24]. Preoperative patient expectations that life will dramatically change after bariatric surgery may have a negative impact on psychological health if these results are not met, even if significant weight loss is achieved. Moreover, some patients realize that certain presurgical problems persist after surgery, which may disappoint them because they cannot attribute underlying emotional disturbance to their weight. Furthermore, patients may have difficulty coping with negative life events that they were previously able to attribute to their obesity.

On the long term, some studies report decreases in levels of depression up to two [29] and even four years postoperatively [30], whereas others report initial improvement followed by decline. The latter seems to correspond to initial weight loss followed by regain or weight stabilization [21]. Initial improvements after surgery may be due to positive reinforcement from frequent post-op clinic visits [31], and as the frequency of followup visits decrease, as does the patient's psychological state. Although more research is needed to determine mental health status several years after bariatric surgery, followup must be conducted long term after surgery to assess and support the patient's psychological well-being.

It is of note that although several studies have reported increased rates of suicide in their patient population after surgery, there is significant variation in cohort characteristics and length of followup in these studies. Thus, it is difficult to adequately compare suicide rates in postbariatric patient populations to the general population [22]. 

### 3.4. Self-Concept and Personality

Self-concept refers to a patient's perception of “self” and includes several important characteristics with respect to the bariatric population: self-esteem, body image, self-confidence, and sense of attractiveness, and assertiveness. Although these factors have not been studied in a standardized or systematic fashion, a review of the literature seems to suggest that weight loss surgery improves self-esteem, self-confidence, and expressiveness [21]. These changes appear to be correlated with major improvements in body image and weight-loss satisfaction after surgery [21]. However, residual body image dissatisfaction due to increasing and/or sagging skin has been reported after surgery in as high as 70% of patients in one particular study [32], even if 90% were pleased with their *overall *appearance. Patients who reported greater satisfaction after surgery were found to have lost less weight than their dissatisfied counterparts, likely because their “skin problems” were less pronounced. Bariatric surgeons must therefore counsel their patients prior to surgery regarding common postoperative skin changes in order to mitigate psychological distress. As patients seek out body-contouring surgery to address skin issues, plastic surgeons also play an important role in discussing the benefits and limitations to plastic surgery. 

No definitive conclusions can be made evaluating the impact of obesity surgery on personality disorders. Favouring improvement, a review noted reduction in neuroticism, defensiveness and immature identity with an increase in discipline in patients after surgery [24]. On the other hand, this review found certain studies that reported no significant changes in personality pathology, perhaps because these disorders manifest early in life and are quite stable and resistant to environmental change. 

### 3.5. Eating Behavior

Eating behavior disorders are fairly common among the obese population, particularly binge eating disorder (BED), which occurs in 5–15% of patients who present for surgery [5]. Postsurgical data evaluating eating behavior is difficult to assess primarily because of variability in the definition of binge eating employed by different studies. This contributes to inconsistencies as to whether psychological aspects of binge eating can be cured by bariatric surgery [33]. Regardless, a review suggests that binge eating behavior may be alleviated after surgery, likely because patients are required to follow strict small meal diets postoperatively [24]. Following this regimen is likely to lead to normalization in eating pattern on the long term. 

However, many patients continue to suffer from psychologically distressing eating behaviors after surgery, specifically those with preoperative eating disorders [24]. This includes rigid eating control due to a continuous fear of weight regain. Although consuming unusually large amounts of food becomes near physically impossible after bariatric surgery, some patients report loss of eating control and claim they would continue to overeat if it were not for early satiety and vomiting. Interestingly, a recent comprehensive interview study of eating behavior two years after surgery identified a subset of patients who reported vomiting for weight and shape reasons [34]. This is clinically important because postoperative vomiting has long been thought of as involuntary and secondary to the physical consequences of surgery.

### 3.6. Pediatric Obesity and Bariatric Surgery

Rates of extreme pediatric obesity are increasing at an alarming rate, and as the prevalence of obesity rises, as does the prevalence of comorbidities such as diabetes, obstructive sleep apnea, steatohepatitis, and cardiac disease. These complications were once thought of as “adult-onset” diseases. With respect to psychosocial comorbidity, it comes as no surprise that that severely obese adolescents are a particularly vulnerable group. Obese children suffer from alienation, poor self-esteem, body dissatisfaction, depressive symptoms, loss-of-control eating, unhealthy weight control behaviors, and impaired social relationships [35, 36]. In one study, HRQoL in severely obese children and adolescents was reported just as low as in children diagnosed with cancer [37]. Finally, the risk of psychological distress appears to increase with age and is greater among girls than boys [38, 39]. 

Initial treatment of pediatric obesity relies on a family-centered approach to improve food quality and physical activity while reducing caloric intake [40]. Although there is limited data evaluating the long-term effects of dietary and behavioral treatments in obese pediatric patients, these interventions generally have poor success. When these interventions are combined with pharmaceuticals, weight management appears to improve [41]. If a severely obese pediatric patient with comorbidities fails initial treatment, bariatric surgery can be considered, even though long-term risks have yet to be determined [42]. A systematic review of the literature reveals that weight loss after surgery in adolescents has been comparable to that in adults, with an average of 50–60% of excess weight lost in the first year and up to 75% by the end of the second year [42]. Absolute BMI reduction in adolescents postsurgery is approximately 35%, leading to improvements and resolution of hypertension, insulin resistance, type 2 diabetes, and dyslipidemia [42].

Current research, though limited, reveals that bariatric surgery may also lead to important psychological benefits in the pediatric population. Recent studies have reported improvements in psychological health (depression, anxiety, and self-concept) after RYGB for patients four months [43] and up to two years postoperatively [44]. As with adults, changes in mental heath seem to parallel weight change and stability, yet a subset of the patient population maintained health gains despite still being overweight or obese. Zeller et al. [44] suggest that a change in weight, reduction of comorbidities, or a patient's revitalized self-concept may override the patient's concern with actual weight status when assessing psychological health. 

## 4. Conclusions

Bariatric surgery is a clinical and cost-effective treatment strategy for the morbidly obese. Although the impact of bariatric surgery on psychological health is often overshadowed by the significant reduction in physical comorbidities, it is important to investigate the former as obesity is strongly correlated with psychological distress and may have a marked effect on quality of life. 

This review reveals overall improvements in psychopathology, depressive symptoms, eating behavior, body image, and HRQoL following bariatric weight loss surgery. In addition, preliminary studies suggest that mental health gains are also achieved in the obese pediatric population. These improvements are most consistent with weight loss and subsequent sequelae such as change in self-esteem, self-concept, and body image. Postoperative psychological health is also influenced by the patient's sense of taking control of their life and by physical and mental support from health care staff.

Not all bariatric patients, however, experience mental health gains from weight loss surgery, which is likely attributable to patients' reactions to common undesired physical outcomes postsurgery: lack of weight loss, weight regain, and undesirable skin changes. Patients' expectations that bariatric surgery will undoubtedly change their life may also set them up for psychological failure if expectations are not met. The health care team must recognize that the negative reactions to adverse events may be accentuated in obese patients seeking surgery as they have a higher prevalence of psychological distress compared to obese individuals in the community. The preoperative evaluation is therefore critical to identify and follow those at risk of persistent or worsening psychopathology after surgery. It will also serve to detail the necessity of postoperative behavioral and lifestyle change and its effect on weight loss. Long-term intensive postoperative followup to evaluate and support lifestyle change can have a tremendous effect on weight loss as well as physical and psychological comorbidities.
